# Racial/ethnic, gender, and age group differences in cardiometabolic risks among adults in a Northern California health plan: a cross-sectional study

**DOI:** 10.1186/s12889-021-11011-4

**Published:** 2021-06-25

**Authors:** Nancy P. Gordon, Loretta Hsueh

**Affiliations:** Division of Research, Kaiser Permanente Northern California, 2000 Broadway, Oakland, CA 94612 USA

**Keywords:** Cardiometabolic risks, Smoking, Diet, Sleep, Racial/ethnic differences in health risks, Gender differences in health risks, Age differences in health risks, African American health, Filipino American health, Latinx health, Chinese American health

## Abstract

**Background:**

In the U.S., the prevalence of diabetes and hypertension are higher among African American/Black (Black), Latinx, and Filipino adults than non-Hispanic White (White) and Chinese adults. We compared the racial/ethnic-specific prevalence of several modifiable cardiometabolic risks in an insured adult population to identify behaviors that may drive racial/ethnic differences in cardiometabolic health.

**Methods:**

This cross-sectional study used data for middle-aged (35–64) and older (65–79) Kaiser Permanente Northern California (KPNC) adult health plan members. Smoking status and BMI were derived from electronic health record data. Weighted pooled self-reported data from the 2014/2015 and 2017 KPNC Member Health Survey cycles were used to estimate daily number of servings of fruits/vegetables, general sodium avoidance, sugar-sweetened beverage (SSB) consumption frequency, alcohol use within daily recommended limit, weekly exercise frequency, and number of hours of sleep daily. Age-standardized estimates of all cardiometabolic risks were produced for middle-aged and older-aged women and men in the five racial/ethnic groups. Analyses focused on racial/ethnic differences within age-gender groups and gender and age group differences within racial/ethnic groups.

**Results:**

In both age groups, Black, Latinx, and Filipino adults were more likely than White and Chinese adults to have overweight and obesity and were less likely to engage in health promoting dietary (fruit/vegetable and SSB consumption, sodium avoidance (women only)) and sleep behaviors. Middle-aged Black and Filipino men were more likely than White men to be current smokers. Less racial/ethnic variation was seen in exercise frequency. Significant gender differences were observed for dietary behaviors overall and within racial/ethnic groups, especially among middle-aged adults; however, these gender differences were smaller for sleep and exercise. Age differences within gender and racial/ethnic groups were less consistent. Racial/ethnic and gender differences in these behaviors were also seen in the subsample of adults with diabetes and/or hypertension and in the subsample of adults who reported they were trying to engage in health promoting behaviors.

**Conclusions:**

Black, Latinx, and Filipino adults were more likely than White and Chinese adults to report dietary and sleep behaviors associated with development and worsening of cardiometabolic conditions, with men exhibiting poorer dietary behaviors than women.

**Supplementary Information:**

The online version contains supplementary material available at 10.1186/s12889-021-11011-4.

## Background

Disparities between African American/Black (Black), Hispanic/Latinx (Latinx), and non-Hispanic White (White) adults in the prevalence of diabetes, hypertension, and obesity have been well-documented, with Black and Latinx populations having a higher prevalence than White populations of all three health conditions [1–5]. The prevalence of these conditions in Filipino Americans (Filipino) and Chinese Americans (Chinese), the two largest Asian ethnic groups in the U.S., and how the prevalence in these ethnic groups compares with each other and with the prevalence among White, Black, and Latinx adults has been less studied [6, 7]. In surveys of U.S. adults and studies based on electronic health records (EHR), Filipino adults have been found to have a higher prevalence than Chinese adults of hypertension [7–10], diabetes [7, 9, 11], coronary heart disease [7, 12], and obesity [7–9, 12], with diabetes and hypertension prevalence among Filipino adults similar to that among Black adults and prevalence among Chinese adults more similar to that among White adults [7].

Adopting healthy lifestyle behaviors, like abstaining from tobacco use, eating a healthy diet (e.g., high consumption of fruits and vegetables, avoidance of high sodium/salty foods and sugary beverages), getting adequate exercise and sleep, and keeping within recommended alcohol intake limits, can reduce the risk of developing hypertension and chronic cardiometabolic (CMB) health conditions [13] and help manage these conditions after onset. Differences in the prevalence of smoking are well documented between White, Black, and Latinx adults, but there is little contemporary information about smoking prevalence among Filipino and Chinese adults. There is also little contemporary information about these other health behavior risks in any of these five racial/ethnic groups. A few studies have shown that compared to White adults, Black and Latinx adults have poorer quality diet [14], and are less likely to meet recommended levels of exercise [15]. Filipino adults have been found to get lower amounts of physical activity than White adults [8] and to have a higher prevalence of smoking than Chinese adults [7]. A survey of Chinese and Filipino adults residing in the Philadelphia, PA, area found that low percentages were following recommended guidelines for fruit and vegetable consumption, use of salt, consumption of sweets, and physical activity [16].

Four factors currently limit our knowledge of how obesity and behavioral health risks for CMB conditions vary across racial/ethnic groups. First, many large studies examining racial/ethnic differences are based on data from nationally representative samples rather than data localized to a region or community. This can result in masking of racial/ethnic differences or limited generalizability to racial/ethnic groups residing in different regions of the U.S. For example, a recent comparison of lifestyle behaviors among different racial/ethnic groups revealed considerable geographic variation in the age-adjusted estimated prevalence of health risks among U.S. adults of different race/ethnicities at the state level [17]. Second, health behaviors and health risks can vary significantly by gender [7, 18–21] and across age groups [20, 22, 23]. Thus, prevalence estimates of obesity, smoking, and other health behavior/lifestyle risks that are not analyzed separately by gender or that are based on a wide age range (e.g., all adults aged 20 years and over, even if age-standardized) may mask important racial/ethnic differences. Third, many studies have examined only one or two CMB risks in a very limited number of racial/ethnic groups. This leads to creation and comparison of behavioral risk profiles for different racial/ethnic groups that are based on very different study populations (e.g., chronic disease patient populations, community-based convenience samples, state and national surveys), modes of data collection, and time periods. Fourth, the prevalence of health behaviors changes over time, but in the absence of more recent data, potentially outdated statistics are used in racial/ethnic group comparisons. For example, prevalence of cigarette smoking among U.S. adults has seen a major decline over the past decade across all racial/ethnic groups [24]. The percentage of U.S. adults who were classified as physically inactive declined by 10 percentage points between 2008 to 2018, while the percentage who met minimum requirements for aerobic physical activity increased by a similar amount during that interval [25]. Consumption of fruits and vegetables has been relatively stable after declining between the 1990s and early 2000s [26], while intake of dietary sugars, especially corn-derived sweeteners, has been increasing [27].

To address these limitations, we conducted a study using contemporary electronic health record and member survey data for a geographically defined sample of insured adults who received healthcare from the same Northern California healthcare delivery system. Our primary aim was to estimate and compare the prevalence of modifiable CMB risks (smoking, weight, dietary behaviors, exercise frequency, sleep adequacy, and alcohol use) in White, Black, Latinx, Filipino, and Chinese American women and men. Our second aim was to examine gender and age group differences in CMB risks within and across these racial/ethnic groups. A third aim was to estimate and compare CMB risks in these same demographic subgroups among those who self-identified as having diabetes and/or hypertension.

## Methods

### Setting

Kaiser Permanente Northern California (KPNC) provides primary and specialty health care to a sociodemographically diverse health plan membership that includes over 3.2 million adults residing mostly in the San Francisco Bay Area, Greater Bay Area, Sacramento area, Silicon Valley, and Central Valley. The KPNC adult membership is similar to the non-Medicaid insured adult population of Northern California with regard to sociodemographic and health characteristics [28].

### Data sources

#### Electronic health record (EHR) cohort

Smoking and weight ranges were estimated from 2016 EHR data. These data came from DECKA2016, an EHR-based cohort created to study racial/ethnic differences in health risks and chronic conditions in a very large (> 2.4 million) cohort of adults aged 20–89 who were health plan members during the entirety of calendar year 2016 [7]. For this study, we restricted data to adults aged 35–79 whose primary language in the EHR was English to parallel the separate member survey cohort. Availability of smoking status and BMI data (requiring a valid weight measured at a calendar year 2016 office visit) varied by race/ethnicity and gender. Smoking status data were available for > 97% (range 90.6–99.2%) of adults aged 35–64 (middle-aged adults) and > 99% (range 97.9–99.5%) of adults aged 65–79 (older-aged adults). Usable BMI data was available for 69.6% (range 65.2–73.4%) of middle-aged adults and 76.7% (range 71.8–80.3%) of older-aged adults.

#### Member health survey (MHS)

The KPNC adult Member Health Survey (MHS) is a self-administered survey (mailed print or online questionnaire) conducted with an age-, gender-, and geographically-stratified sample of health plan members aged 20 years and over who were members at the time of the survey (Spring of a survey year) and during at least the fourth quarter of the preceding year. The MHS is only conducted in English. The survey collects information on sociodemographic characteristics and a wide range of variables related to health, including those used for the current study. Survey details are published elsewhere [29]. For our MHS-based analyses, we used pooled survey data obtained from the 2014/2015 and 2017 MHS cycles for men and women aged 35–79, restricted to our five racial/ethnic groups. The overall response rate for ages 35–64 was 35 and 64% for ages 65 to 79, with slightly lower response rates for Black, Latinx, and Filipino adults and younger men.

The KPNC Institutional Review Board approved the analyses based on the MHS and DECKA2016 cohort data undertaken for this report.

### Study sample

Of the 1,387,569 adults from the DECKA2016 study sample used to estimate smoking prevalence, 73.4% were middle-aged (35–64 years). In the middle-aged group (*n* = 1,018,012), 60.3% (*n* = 613,854) were White, 9.8% (*n* = 99,492) were Black, 16.0% (*n* = 162,752) were Latinx, 8.3% (*n* = 84,413) were Filipino, and 5.6% (*n* = 57,501) were Chinese adults. The older-aged group (65–79 years) used to estimate smoking prevalence was 73.1% (*n* = 270,153) White, 8% (*n* = 29,414) Black, 8.6% (*n* = 32,015) Latinx, 6.2% (*n* = 22,826) Filipino, and 4.1% (*n* = 15,149) Chinese adults. The DECKA2016 sample used to classify BMI ranges was smaller (726,079 middle-aged adults and 285,729 older-aged adults) due to inability to calculate a 2016 BMI. By age group, the middle-aged sample was 60.0% (*n* = 435,610) White, 9.5% (*n* = 68,788) Black, 16.2% (*n* = 117,838 Latinx), 8.6% (*n* = 62,470 Filipino), and 5.7% (*n* = 41,373) Chinese adults, and the older-aged sample was 72.8% (*n* = 208,017) White, 7.6% (*n* = 21,704) Black, 8.9% (*n* = 25,495) Latinx, 6.4% (*n* = 18,309) Filipino, and 4.3% (*n* = 12,204) Chinese adults. In all racial/ethnic groups, the ratio of women to men was approximately 54%:46%.

The MHS survey sample included 15,203 adults aged 35–79. Race/ethnicity was self-reported. Among middle-aged respondents (*n* = 9067), 53.4% (*n* = 4841) were White, 12.5% (*n* = 1131) were Black, 19.6% (*n* = 1780) were Latinx, 7.3% (*n* = 658) were Filipino, and 7.2% (*n* = 657) were Chinese. In the 65–79 age group (*N* = 6136), 68.2% (*n* = 4185) were White, 9.3% (*n* = 574) were Black, 11.7% (*n* = 716) were Latinx, 6.0% (*n* = 368) were Filipino, and 4.8% (*n* = 293) were Chinese. In both age groups, gender distribution within racial/ethnic groups was approximately equal. Survey weights were applied in the analysis to adjust the age and gender composition of each racial/ethnic group to the age and gender composition of English-speaking members in the corresponding racial/ethnic group in the DECKA2016 cohort that was used for the smoking and weight estimates.

### Study variables

#### Weight range

Weight ranges were based on BMI (calculated from the clinic-measured weight closest to December 1, 2016 and the clinic-measured height closest to that date. We used standard BMI thresholds to create weight ranges for White, Black, and Latinx adults (BMI of 18.5 - < 25 kg/m^2^ for healthy weight, > 25 kg/m^2^ for overweight/obese, and ≥ 30 kg/m^2^ for obese) and WHO-recommended [30] lower thresholds for Asians for the Filipino and Chinese adults (BMI of 18.5 - < 23 kg/m^2^ for healthy weight, > 23.7 kg/m^2^ for overweight/obese, and ≥ 27.5 kg/m^2^ for obese).

#### Smoking status

Adults were categorized as being current, former, or never smokers based on smoking information in the EHR for the visit date closest to December 1, 2016. Those who did not have smoking status ascertained at a clinic visit in 2015 or 2016 but who were coded as never smokers at visits during 2012–2014 or January–March 2017 were classified as never smokers.

#### Alcohol use

Alcohol use was assessed using the survey question “On days when you had a drink, how many drinks did you usually have?”. All women and men who reported not having had a drink in the prior 12 months, women and men aged < 66 who indicated having ≤1 drink, and men aged ≥66 who reported having ≤2 drinks were coded as keeping within recommended daily limit [31].

#### Dietary behaviors

The survey asked about three dietary behaviors: daily fruit and vegetable intake, avoidance of high sodium foods, and frequency of sugar-sweetened beverage (SSB) consumption. Daily fruit and vegetable intake was assessed using the question “During an average day, about how many servings of fruits and vegetables do you usually eat? *(1 serving = a half cup or a medium piece).*” Responses were coded to reflect ≥3 servings and ≥ 5 servings a day. Avoidance of high sodium foods was based on the question “How often do you try to avoid eating foods that are high in salt or sodium (like most canned, packaged, processed, and “fast“ foods and foods seasoned with a lot of salt)?” People who indicated “most” or “all” of the time were classified as “Tries to avoid high sodium/high salt foods most or all of the time.” Frequency of SSB consumption was assessed with the question “How many days per week do you usually drink one or more sugar- or corn syrup-sweetened drinks like regular soda, fruit drinks, vitamin water, bottled teas, coffee drinks, sports drinks (e.g., Gatorade), and energy drinks (e.g., Red Bull)? Do not count diet drinks***.***” People were classified as consuming SSBs ≥5 days a week, ≥ 3 days a week, and < once a week or never.

#### Exercise/physical activity frequency

Exercise or physical activity frequency was assessed using the survey question “How often do you usually do physical activity or exercise (such as walking, running, swimming, tennis, soccer, gardening, dancing, yoga, exercise class, etc.)?” Adults were classified as getting exercise ≥5 days a week, ≥ 3 days a week, and < once a week.

#### Sleep behavior

Sleep behavior was assessed using the survey question “On a typical weekday, how many total hours of sleep do you usually get, including naps?” Because the American Academy of Sleep Medicine (AASM) and Sleep Research Society (SRS) both recommend that all adults get ≥7 h of sleep a day [32], we used that as our measure of getting adequate sleep, with no upper limit placed on amount of sleep as incorporated into National Sleep Foundation recommendations [33]. We also classified people as getting < 7 h a day, which the AASM and SRS consider insufficient sleep, and <  6 h of sleep a day, which is considered to be “short sleep” [34].

#### Self-reported efforts to engage in health-promoting behaviors

In response to the question “Are you currently doing any of the following to improve or maintain your health?”, respondents could endorse “Try to eat mostly healthy foods”, “Get moderate or vigorous exercise most days” and/or “take walks for at least 30 minutes most days” (subsequently combined into a single variable “Try to get exercise most days”), and “Try to get enough sleep to feel well rested.”

### Statistical analysis

Data were analyzed using SAS version 9.4 (SAS Institute, Cary, NC, 2013) [35]. Data were analyzed separately for men and women based on EHR-documented gender. Unweighted data from DECKA2016 were used for the weight and smoking analyses. Survey data were weighted to reflect the age (5-year intervals) group by gender composition of respondents’ corresponding racial/ethnic groups based on the DECKA2016 race/ethnicity cohort. To facilitate direct comparison of prevalence estimates across racial/ethnic groups, and by gender within racial/ethnic groups, we age-standardized all prevalence estimates based on the DECKA2016 cohort and weight survey data to the 2016 Census (American Community Survey). Prevalence estimates for the middle-aged group were standardized to the following age distribution: 35–44 years, 45.46%; 45–54 years, 31.92%; 55–64 years, 22.62%. Estimates for the older-aged group were standardized as follows: 65–69 years, 32.48%; 70–74 years, 34.30%; 75–79 years, 33.22%. Supplemental Table 1 shows the age distributions of the middle- and older-aged study groups prior to age-standardization.

All age-standardized prevalence estimates and 95% confidence intervals (CIs) were produced using the Proc Surveyreg procedure recommended by the Centers for Disease Control and Prevention [36]. For the two age groups, we ran separate sets of Proc Surveyreg models to produce race/ethnicity-specific prevalence estimates, using gender as a domain. Estimate statements in the models were used to assess whether differences between the age-standardized prevalence estimates for White adults versus each of the other four racial/ethnic groups and Filipino versus Chinese adults were statistically significant within that age-gender group. Due to large sample sizes for the smoking and weight data, we used non-overlapping 95% CIs as an indicator that age-standardized estimates significantly differed by gender within the same racial/ethnic and age group. For the survey-derived health behaviors, we used Proc Surveylogistic models with weighted survey data to examine whether there were gender differences within the same racial/ethnic group after adjusting for age using the same age groups as were employed for the age-standardization (ages 35–44 years, 45–54, 55–64, and 65–69, 70–74, 75–79, respectively, for ages 35–64 and 65–79). Proc Surveylogistic models were also used to evaluate differences by age group (middle-aged vs. older-aged) within racial/ethnic-gender groups.

We also conducted two additional sets of analyses to produce and compare age-standardized prevalence estimates of selected survey-derived health behaviors for Black, Latinx, Filipino and Chinese men and women to those of White adults. For these analyses, we compared adults ages 35–79 in these racial/ethnic groups and used the same Proc Surveyreg procedure as previously described with the following age-standardization distribution: 35–44, 24.17%; 45–54 years, 25.53%, 55–64 years, 24.73%; 65–74 years, 17.08%; and 75–79, 8.49%. The first set of analyses was restricted to adults who reported that they were trying to engage in healthy behaviors. Among those who indicated that they tried to eat mostly healthy foods, we compared the percentages who reported consuming ≥3 servings of fruits and vegetables a day, who tried to avoid high sodium/salty foods most or all of the time, and who consumed sugary beverages ≥5 days a week, ≥ 3 days a week, and ≤ once/week. Among those who indicated that they got exercise most days, we compared the percentages of women and men in the four non-White racial/ethnic groups who got exercise ≥5 days a week and ≥ 3 days a week. Among those who indicated that they tried to get enough sleep to feel well-rested, we compared the percentages who reported getting ≥7 h of sleep, < 7 h of sleep, and <  6 h of sleep a day. The second set of analyses compared racial/ethnic groups on prevalence of survey-based health behaviors restricted to adults who self-reported having diabetes and/or hypertension.

## Results

Table 1 presents provides a description of the characteristics of the middle-aged and older-aged women and men in the five racial/ethnic groups based on the survey data. Briefly, higher percentages of Black and Latinx adults in both age groups had lower educational attainment and household income than White and Chinese adults. Filipino adults were more likely than Black and Latinx adults, but less likely than Chinese adults, to be college graduates. In the older-aged group, Filipino adults were more likely than Chinese and White adults to have a lower household income. In terms of health-related factors, Black and Filipino adults in both age groups, and Latinx adults in the older-aged group, were more likely than White adults to report having diabetes and/or hypertension. In both age groups, Chinese adults did not significantly differ from White adults in prevalence of diabetes and/or hypertension, but had a significantly lower prevalence of diabetes and/or hypertension than Filipino adults. Black and Latinx women and men in both age groups and Filipino men in the older-aged group were more likely than White adults of each corresponding gender group to report being in fair or poor health. Among women in both age groups and men in the middle-aged group, Black, Latinx, Filipino, and Chinese adults were more likely than White adults to believe that their weight, diet, exercise, and other behaviors had little to only moderate effect on their health; among men in the older-aged group, White, Black, and Chinese men did not significantly differ, but Latinx and Filipino men were more likely to hold this belief.

**Table 1:** Characteristics of racial/ethnic groups ages 35–64 and 65–79, by gender, after weighting and age-standardization

	Women					Men				
	White	Black	Latina	Filipina	Chinese	White	Black	Latino	Filipino	Chinese
	%	%	%	%	%	%	%	%	%	%
**Ages 35–64**										
Education										
≤ High school graduate	14.7	17.8	31.4	11.5	5.2	18.6	29.4	37.4	12.9	7.9
Some college/AA degree	33.8	43.8	38.0	32.3	23.4	28.9	38.5	35.3	38.3	18.8
College graduate	51.5	38.4	30.6	56.2	71.4	52.5	32.1	27.3	48.8	73.3
Household income										
≤ $35,000	10.1	19.8	16.9	9.7	6.5	7.7	19.8	11.3	7.6	4.1
$35,001 - < $65,000	16.9	26.0	26.5	24.1	16.3	14.0	20.5	22.2	20.8	12.1
$65,001 - < 80,000	12.9	13.5	14.2	13.6	10.3	9.2	10.6	15.2	10.1	11.0
≥ $80,000	60.1	40.7	42.4	52.6	66.9	69.1	49.1	51.3	61.5	72.8
Health status										
Very good or excellent	61.9	41.2	52.1	54.5	55.4	58.1	44.6	46.0	52.1	57.2
Good	30.6	43.0	37.1	36.5	35.8	32.1	39.5	40.7	36.6	35.1
Fair or poor	7.5	15.8	10.8	9.0	8.8	9.8	15.9	13.3	11.3	7.7
Has diabetes and/or hypertension	20.6	45.7	23.3	39.5	21.8	26.7	42.0	33.5	48.5	22.6
Believes health behaviors/ lifestyle have no, little or only moderate effect on health	5.2	10.8	9.3	23.1	12.9	9.1	15.3	16.5	21.1	16.8
**Ages 65–79**										
Education										
≤ High school graduate	24.0	34.6	50.8	19.3	18.3	19.3	29.9	33.4	18.2	16.1
Some college/AA degree	37.1	38.4	33.6	22.0	34.0	31.2	37.9	39.1	34.5	21.9
College graduate	38.9	27.0	15.6	58.7	47.7	49.5	32.2	27.5	47.3	62.0
Household income										
≤ $35,000	26.1	37.9	42.4	37.4	16.1	14.9	21.0	19.2	36.4	17.6
$35,001 - < $65,000	28.1	28.3	32.8	28.3	24.4	24.1	28.3	31.9	30.4	20.4
$65,001 - < 80,000	13.0	13.2	10.9	9.8	11.9	14.2	13.2	12.3	8.0	15.6
≥ $80,000	32.8	20.6	13.8	24.5	47.6	46.8	37.5	36.6	25.2	46.4
Health status										
Very good or excellent	50.1	28.3	36.4	32.4	41.9	47.5	32.6	40.9	32.2	47.3
Good	36.5	49.0	40.1	48.9	58.1	38.0	44.4	39.6	45.7	42.6
Fair or poor	13.4	22.7	23.5	18.7	10.0	14.5	23.0	19.5	22.1	10.1
Has diabetes and/or hypertension	53.5	72.6	65.6	78.8	53.1	58.9	77.7	65.2	75.3	54.7
Believes health behaviors/ lifestyle have no, little or only moderate effect on health	7.3	13.7	19.9	38.8	22.3	12.1	15.3	21.7	43.5	17.1

All percentages are based on self-reported data from the pooled 2014/2015 and 2017 Member Health Surveys weighted to the age-gender composition of KPNC adult members in the relevant race/ethnic group whose preferred spoken language was English and then standardized to the age distribution of 35–64 and 65–79 year old adults in the U.S. population in 2016. White: non-Hispanic White; Black: African American/Black

Table 2 (middle-aged adults) and Table 3 (older-aged adults) show prevalence of weight ranges and health behaviors for men and women in the five racial/ethnic groups. Table 4 summarizes differences in weight ranges and health behaviors across Black, Latinx, Filipino, and Chinese adults compared to White adults and for Filipino adults compared to Chinese adults.

**Table 2:** Differences in weight and health behavior risks, White, Black, Latinx, Filipino, and Chinese women and men aged 35–64 years

	Women					Men				
Weight and Behavior Risks	White	Black	Latina	Filipina	Chinese	White	Black	Latino	Filipino	Chinese
	% (95% CI)	% (95% CI)	% (95% CI)	% (95% CI)	% (95% CI)	% (95% CI)	% (95% CI)	% (95% CI)	% (95% CI)	% (95% CI)
**Weight** ^1^										
Healthy weight	34.3 ^a^ (34.1–34.5)	14.6 ^ab^ (14.2–14.9)	20.4 ^ab^ (20.1–21.0)	20.6 ^abc^ (20.2–21.0)	44.5 ^ab^ (43.9–45.2)	18.3 (18.2–18.5)	13.4 ^d^ (13.0–13.8)	9.7 ^b^ (9.5–10.0)	7.2 ^bc^ (6.9–7.6)	18.7 (18.1–19.3)
Overweight or obese	64.6 ^a^ (64.4–64.8)	85.0 ^b^ (84.6–85.3)	79.2 ^ab^ (78.9–79.5)	78.6 ^abc^ (78.2–79.0)	51.7 ^ab^ (51.0–52.3)	81.4 (81.3–81.6)	86.4 ^b^ (86.0–86.8)	90.2 ^b^ (90.0–90.5)	92.5 ^bc^ (92.2–92.9)	80.8 (80.2–81.4)
Obese	35.1 ^a^ (34.9–35.3)	57.6 ^ab^ (57.1–58.0)	47.7 ^ab^ (47.3–48.1)	38.1 ^abc^ (37.6–38.6)	16.4 ^ab^ (15.9–16.9)	39.6 (39.4–39.8)	50.4 ^b^ (49.8–51.0)	51.7 ^b^ (51.2–52.1)	51.8 ^bc^ (51.2–52.4)	29.7 ^b^ (29.0–30.3)
**Smoking status**										
Never smoker	68.3 ^a^ (68.2–68.5)	69.3 ^a^ (69.0–69.7)	77.5 ^ab^ (77.3–77.8)	81.2 ^abc^ (80.9–81.6)	92.0 ^ab^ (91.7–92.8)	63.4 (63.2–63.6)	62.8 (62.3–63.3)	66.6 ^b^ (66.3–67.0)	55.6 ^bc^ (55.1–56.1)	77.2 ^b^ (76.7–77.8)
Former smoker	23.5 (23.3–23.6)	19.7 (19.3–20.0)	17.4 (17.1–17.7)	14.1 (13.8–14.4)	6.3 (6.0–6.6)	24.6 (24.4–24.7)	21.2 (20.8–21.6)	23.4 (23.1–23.7)	31.0 (30.5–31.5)	15.7 (15.2–16.1)
Current smoker	8.2 ^a^ (8.1–8.3)	11.0 ^ab^ (10.8–11.1)	5.1 ^ab^ (4.9–5.2)	4.7 ^abc^ (4.5–4.8)	1.7 ^ab^ (1.6–1.9)	12.0 (11.9–12.1)	16.0 ^b^ (15.7–16.4)	10.0 ^b^ (9.7–10.2)	13.4 ^bc^ (13.0–13.7)	7.1 ^b^ (6.8–7.4)
**Alcohol use**										
Stays within recommended daily limit^2^	61.1 ^a^ (59.1–63.1)	68.2 ^ae^ (64.3–72.1)	62.1 ^a^ (58.8–65.4)	84.3 ^b^ (80.4–88.2)	89.5 ^ab^ (86.0–93.0)	78.5 (76.6–80.3)	79.8 (75.8–83.7)	68.0 ^b^ (64.6–71.5)	81.0 ^c^ (76.0–86.1)	96.8 ^b^ (94.7–98.9)
**Dietary behaviors**										
Fruit and vegetable intake										
≥ 3 servings a day	67.7 ^a^ (65.8–69.5)	41.2 ^ab^ (37.3–45.1)	41.8 ^ab^ (38.6–44.9)	26.2 ^bc^ (21.8–30.6)	53.9 ^ab^ (48.5–59.3)	46.9 (44.7–49.1)	23.0 ^b^ (19.0–27.0)	29.6 ^b^ (26.3–32.8)	19.6 ^bc^ (14.7–24.4)	36.7 ^b^ (31.3–42.1)
≥ 5 servings a day	25.3 ^a^ (23.6–27.0)	11.4 ^ab^ (8.8–13.9)	11.7 ^ab^ (9.7–13.8)	6.4 ^bc^ (3.9–8.9)	14.3 ^b^ (10.5–18.1)	14.2 (12.7–15.7)	4.9 ^b^ (3.0–6.8)	7.0 ^b^ (5.1–8.8)	3.9 ^bc^ (1.5–6.3)	12.7 (9.0–16.5)
Tries to avoid high sodium/high salt foods most or all of the time	65.9 ^a^ (64.1–67.8)	58.7 ^b^ (54.8–62.6)	59.5 ^ab^ (56.4–62.6)	49.9 ^bc^ (44.9–54.8)	61.4 ^a^ (56.2–66.6)	53.4 (51.3–55.6)	54.7 (50.1–59.3)	52.7 (49.1–56.2)	46.5 ^d^ (40.5–52.6)	48.7 (43.1–54.2)
Frequency consumes sugar-sweetened beverages^3^										
< Once a week or never	61.5 ^a^ (59.0–64.1)	36.1 ^ab^ (31.7–40.4)	48.8 ^ab^ (45.1–52.5)	46.0 ^abc^ (39.8–52.2)	57.4 ^a^ (50.6–64.2)	45.6 (42.0–48.4)	28.5 ^b^ (23.7–33.3)	31.4 ^b^ (27.6–35.2)	28.5 ^bc^ (21.5–35.5)	43.9 (37.2–50.6)
≥ 3 days a week	25.1 ^a^ (22.8–27.4)	46.9 ^b^ (42.4–51.5)	34.8 ^ab^ (31.3–38.4)	40.8 ^abc^ (34.7–47.0)	25.0 (19.1–31.0)	35.2 (32.5–37.9)	51.2 ^b^ (45.9–56.5)	49.3 ^b^ (45.2–53.4)	55.7 ^bc^ (48.0–63.4)	32.0 (25.6–38.4)
≥ 5 days a week	19.1 ^a^ (17.0–21.1)	30.1 ^ab^ (25.9–34.3)	27.3 ^ab^ (24.0–30.7)	33.4 ^bc^ (27.5–39.3)	16.9 (11.7–22.1)	27.1 (24.6–29.7)	37.8 ^b^ (32.7–43.0)	35.5 ^b^ (31.6–39.4)	42.2 ^bc^ (34.6–49.8)	23.2 (17.5–29.0)
Reports trying to eat mostly healthy foods	81.7 ^a^ (80.2–83.2)	72.7 ^b^ (69.2–76.2)	74.7 ^ab^ (71.9–77.5)	74.2 ^e^ (69.9–78.4)	80.1 ^a^ (75.8–84.4)	71.0 (69.1–73.0)	68.6 (64.3–72.9)	69.3 (66.0–72.5)	69.0 (63.3–74.6)	67.7 (62.5–72.9)
**Exercise/physical activity frequency**										
≥ 3 days a week	74.6 (72.9–76.3)	66.4 ^ab^ (62.7–70.2)	71.7 (68.8–74.6)	67.2 ^e^ (62.6–71.9)	69.3 ^d^ (64.4–74.2)	74.1 (72.2–76.0)	72.6 (68.5–76.7)	72.5 (69.4–75.7)	70.2 (64.7–75.7)	67.3 ^d^ (52.1–72.5)
≥ 5 days a week	41.3 (39.3–43.2)	30.3 ^ab^ (26.6–33.9)	35.5 ^e^ (32.4–38.5)	33.3 ^ce^ (28.6–38.0)	40.4 (35.1–45.6)	43.8 (41.7–46.0)	38.9 (34.5–43.4)	39.1 ^d^ (35.7–42.6)	40.7 (34.8–46.6)	34.6 ^d^ (29.4–39.8)
< once a week	10.1 (8.9–11.3)	15.8 ^b^ (12.9–18.7)	11.6 (9.6–13.7)	12.3 (9.0–15.6)	9.7 (6.5–12.9)	8.8 (7.6–10.1)	13.9 ^e^ (10.7–17.2)	11.1 (8.9–13.3)	11.2 (7.4–15.0)	13.5 ^d^ (9.7–17.3)
Reports trying to get exercise most days	65.6 (63.8–67.5)	63.3 (59.5–67.1)	66.8 (63.8–69.9)	74.2 ^bc^ (69.8–78.5)	63.4 (58.2–68.6)	64.1 (62.0–66.2)	68.9 ^d^ (64.6–73.1)	65.8 (62.5–69.2)	72.9 ^ce^ (67.5–78.3)	65.2 (59.9–70.5)
**Usual amount of sleep**										
≥ 7 h a day	70.4 ^a^ (66.6–72.2)	45.7 ^b^ (41.7–49.7)	59.8 ^b^ (56.7–63.0)	51.8 ^bc^ (46.8–56.7)	59.5 ^b^ (54.3–64.7)	66.5 (64.5–68.6)	42.6 ^b^ (38.1–47.2)	56.0 ^b^ (52.4–59.5)	47.2 ^bc^ (41.1–53.3)	64.2 (58.9–69.6)
< 7 h a day	29.6 ^a^ (27.8–31.4)	54.3 ^b^ (50.3–58.3)	40.2 ^b^ (37.0–43.4)	48.2 ^bc^ (43.3–53.2)	40.5 ^b^ (35.3–45.8)	33.5 (31.4–35.5)	57.4 ^b^ (52.8–62.0)	44.0 ^b^ (40.5–47.6)	52.8 ^bc^ (46.7–59.0)	35.8 (30.4–41.1)
< 6 h a day	7.6 (6.6–8.6)	24.8 ^b^ (21.4–28.3)	14.5 ^b^ (12.2–16.8)	18.4 ^b^ (14.5–22.2)	13.0 ^e^ (9.3–16.7)	8.4 (7.1–9.6)	22.7 ^b^ (18.8–26.6)	12.5 ^e^ (10.2–14.9)	18.1 ^bc^ (13.4–22.7)	9.4 (6.2–12.6)
Reports trying to get enough sleep to feel well-rested	76.4 ^a^ (74.7–78.1)	68.0 ^b^ (64.3–71.6)	68.2 ^b^ (65.3–71.2)	73.1 (68.7–77.4)	67.5 ^b^ (62.4–72.5)	67.5 (65.5–69.6)	61.9 ^d^ (57.4–66.3)	65.3 (62.0–68.7)	66.3 (60.6–72.0)	60.5 ^d^ (55.1–66.0)

Estimates for smoking status and weight categories are based on electronic health record (EHR) data for a very large cohort of adults who were KPNC members during all of calendar year 2016. Estimates for other behaviors are based on pooled data from the 2014/2015 and 2017 Member Health Surveys weighted to the age-gender composition of KPNC adult members in the relevant race/ethnic group whose preferred spoken language in the EHR was English. All estimates are standardized to the age distribution of 65–79 year old adults (using age categories 65–69, 70–74, 75–79) in the U.S. population in 2016

^1^ White, Black, and Latinx adults: Healthy weight = BMI 18.5- < 27.5; Overweight or obese = BMI ≥ 27.5; Overweight = BMI 27.5 - < 30.0; Obese = BMI ≥ 30.0; Filipino and Chinese adults: Healthy weight = BMI 18.5 - < 23.0; Overweight or obese = BMI ≥ 23.0; Overweight = BMI 23 - < 27.5; Obese = BMI ≥ 27.5

^2^ Recommended limits: ≤ 1 drink per day for all women and for men aged ≤ 65; ≤ 2 drinks per day for men > age 65

^3^ Based on weighted pooled data from the 2015 and 2017 surveys

^a^ Within racial/ethnic group, women significantly different from men at *p* < .05

^b^ Significantly different from Whites at *p* < .001

^c^ Filipinos significantly different from Chinese at *p* < .05

^d^ Significantly different from Whites at *p* < .05

^e^ Significantly different from Whites at *p* < .01

^f^ Lower bound of 95% CI is < 0

**Table 3:** Differences in weight and health behavior risks, White, Black, Latinx, Filipino, and Chinese women and men aged 65–79 years

	Women					Men				
Weight and Behavior Risks	White	Black	Latina	Filipina	Chinese	White	Black	Latino	Filipino	Chinese
	% (95% CI)	% (95% CI)	% (95% CI)	% (95% CI)	% (95% CI)	% (95% CI)	% (95% CI)	% (95% CI)	% (95% CI)	% (95% CI)
**Weight** ^1^										
Healthy weight	32.8 ^a^ (32.5–33.1)	17.7 ^ab^ (17.0–18.3)	23.2 ^ab^ (22.5–23.9)	23.3 ^abc^ (22.5–24.1)	41.7 ^ab^ (40.5–43.0)	21.2 (21.0–21.5)	21.2 (20.3–22.0)	15.8 ^b^ (15.2–16.5)	13.2 ^bc^ (12.4–13.9)	23.9 ^b^ (22.8–25.0)
Overweight or obese	62.3 ^a^ (65.0–65.5)	81.2 ^ab^ (80.5–81.9)	75.9 ^ab^ (75.2–76.6)	74.8 ^abc^ (73.9–75.6)	52.4 ^ab^ (51.2–53.7)	78.4 (78.2–78.7)	77.8 (77.0–78.7)	83.9 ^b^ (83.3–84.6)	86.2 ^bc^ (85.5–87.0)	74.6 ^b^ (73.5–75.8)
Obese	32.8 (32.5–33.1)	49.7 ^ab^ (48.8–50.6)	41.0 ^b^ (40.2–41.8)	30.7 ^abc^ (29.8–31.6)	17.1 ^ab^ (16.1–18.0)	34.7 (34.4–35.0)	37.9 ^b^ (36.9–38.9)	40.1 ^b^ (39.2–41.0)	38.3 ^bc^ (37.2–39.4)	23.3 ^b^ (22.2–24.4)
**Smoking status**										
Never smoker	57.0 ^a^ (56.7–57.2)	56.5 ^a^ (55.8–57.2)	68.1 ^ab^ (67.4–68.8)	86.7 ^ab^ (86.1–87.3)	88.5 ^ab^ (87.2–89.3)	47.8 (47.5–48.1)	43.0 ^b^ (42.1–43.8)	48.5 (47.7–49.3)	42.0 ^bc^ (41.0–43.0)	70.0 ^b^ (68.9–71.1)
Former smoker	36.7 (36.5–37.0)	34.9 (34.2–35.7)	27.2 (26.5–27.8)	10.6 (10.1–11.1)	9.9 (9.2–10.5)	45.4 (45.1–45.7)	46.5 (45.6–47.4)	45.1 (44.2–45.9)	50.6 (49.6–51.6)	26.5 (25.5–27.6)
Current smoker	6.3 (6.1–6.4)	8.6 ^b^ (8.1–9.0)	4.7 ^ab^ (4.4–5.0)	2.7 ^ab^ (2.4–3.0)	1.6 ^ab^ (1.3–1.9)	6.8 (6.6–6.9)	10.5 ^b^ (10.0–11.1)	6.4 (6.0–6.8)	7.4 (6.9–8.0)	3.5 ^b^ (3.0–3.9)
**Alcohol use**										
Stays within recommended daily limit^2^	75.7 ^a^ (73.5–77.8)	82.3 ^ad^ (77.4–87.3)	83.2 ^ae^ (78.6–87.8)	> 99.9 ^ab^ (−-) ^f^	96.3 ^ab^ (93.1–99.5)	60.1 (57.7–62.6)	64.9 (57.9–72.0)	59.1 (52.9–65.3)	86.5 ^b^ (80.1–92.8)	84.7 ^b^ (77.0–91.7)
**Dietary behaviors**										
Fruit and vegetable intake										
≥ 3 servings a day	62.2 ^a^ (59.9–64.5)	37.4 ^ab^ (31.6–43.1)	38.3 ^ab^ (32.7–43.9)	25.6 ^bc^ (18.8–32.3)	56.1 ^a^ (47.6–64.6)	40.6 (38.3–43.0)	23.3 ^b^ (17.3–29.4)	23.5 ^b^ (18.5–28.6)	17.7 ^bc^ (11.0–24.4)	28.3 ^e^ (20.1–36.4)
≥ 5 servings a day	19.8 ^a^	9.2 ^b^ (5.7–12.7)	11.5 ^ab^ (7.9–15.3)	3.2 ^bc^ (0.7–5.7)	14.5 (8.5–20.5)	12.1 (10.5–13.7)	4.8 ^b^ (1.9–7.6)	2.5 ^b^ (0.8–4.2)	1.8 ^bc^ (−-) ^f^	7.1 ^d^ (2.4–11.8)
	(17.9–21.7)									
Tries to avoid high sodium/high salt foods most or all of the time	66.9 ^a^ (64.7–69.2)	68.4 (62.9–73.9)	59.0 ^e^ (53.3–64.6)	51.9 ^b^ (44.2–59.7)	61.3 (52.8–69.7)	57.5 (55.2–59.9)	64.2 (57.6–70.9)	58.9 (53.0–64.8)	54.9 (46.7–63.0)	55.3 (46.2–64.3)
Frequency consumes sugar-sweetened beverages^3^										
< Once a week or never	70.2 ^a^ (67.3–73.1)	35.3 ^b^ (28.6–42.1)	56.7 ^ab^ (50.0–63.4)	48.2 ^b^ (38.1–58.3)	58.8 ^d^ (48.4–69.2)	57.7 (54.5–60.9)	33.5 ^b^ (25.6–41.4)	40.0 ^b^ (33.3–46.8)	44.0 ^d^ (32.8–55.1)	48.3 (37.4–59.2)
≥ 3 days a week	21.5 ^a^ (18.9–24.2)	48.5 ^b^ (41.5–55.6)	31.9 ^ae^ (26.7–38.1)	41.9 ^bc^ (32.0–51.8)	25.4 (16.2–34.7)	30.4 (27.4–33.4)	49.8 ^b^ (41.5–58.1)	44.0 ^b^ (37.2–50.8)	44.6 ^d^ (33.5–55.8)	38.2 (27.6–48.9)
≥ 5 days a week	16.9 ^a^ (14.5–19.3)	36.7 ^b^ (29.8–43.5)	24.4 ^ad^ (18.7–30.2)	31.5 ^e^ (22.3–40.8)	19.7 (11.2–28.1)	22.8 (20.3–25.7)	31.7 ^d^ (24.1–39.4)	35.7 ^b^ (29.2–42.1)	38.0 ^e^ (27.1–48.8)	31.3 (21.1–41.5)
Reports trying to eat mostly healthy foods	79.9 ^a^ (78.0–81.7)	78.2 (73.3–83.0)	74.6 (69.3–79.4)	80.0 (74.0–86.0)	83.9 ^a^ (77.6–90.2)	70.8 (68.7–73.0)	73.9 (68.1–79.8)	70.7 (65.5–76.0)	74.8 (67.9–81.8)	70.0 (61.8–78.0)
**Exercise/physical activity frequency**										
≥ 3 days a week	75.2 ^a^ (73.2–77.3)	64.0 ^ab^ (58.3–69.7)	68.4 ^ad^ (63.1–73.7)	77.2 ^c^ (70.8–83.6)	86.8 ^b^ (81.1–92.5)	79.6 (77.7–81.5)	74.5 (68.7–80.2)	76.6 (71.6–81.6)	76.6 (69.5–83.7)	83.3 (76.4–90.1)
≥ 5 days a week	45.3 ^a^ (42.9–47.7)	27.3 ^ab^ (22.0–32.6)	39.0 ^ad^ (33.4–44.6)	32.3 ^abc^ (25.0–39.6)	52.3 (43.9–60.6)	53.1 (50.7–55.5)	37.1 ^b^ (30.5–43.8)	48.0 (42.0–53.9)	48.3 (40.1–56.6)	52.0 (42.8–61.2)
< once a week	13.1 ^a^ (11.6–14.7)	15.8 (11.5–20.1)	15.0 (10.9–19.1)	8.3 ^d^ (4.0–12.5)	4.2 ^b^ (0.8–7.6)	8.9 (7.6–10.2)	11.0 (6.8–15.2)	10.8 (7.2–14.5)	11.5 (6.0–16.9)	7.4 (2.6–12.2)
Reports trying to get exercise most days	61.2 (58.9–63.5)	55.8 (50.0–61.6)	55.9 ^a^ (50.2–61.5)	73.3 ^b^ (66.6–80.1)	77.7 ^b^ (70.7–84.7)	64.7 (62.4–67.0)	62.1 (55.6–68.5)	72.1 ^d^ (66.9–77.4)	77.3 ^b^ (70.6–84.1)	71.6 (63.5–79.8)
**Usual amount of sleep**										
≥ 7 h a day	74.7 ^a^ (72.7–76.8)	61.9 ^b^ (56.0–67.8)	65.3 ^e^ (59.7–70.9)	48.3 ^ab^ (40.4–56.2)	52.2 ^b^ (44.4–60.1)	82.1 (80.2–83.9)	60.0 ^b^ (53.1–67.0)	70.0 ^b^ (64.4–75.6)	64.6 ^b^ (56.7–72.6)	66.7 ^b^ (58.3–75.0)
< 7 h a day	25.3 ^a^ (23.2–27.4)	38.1 ^b^ (32.2–44.0)	34.7 ^e^ (29.1–40.4)	47.8 ^b^ (39.9–55.6)	40.8 ^b^ (32.3–49.4)	17.9 (16.1–19.8)	40.0 ^b^ (33.0–46.9)	30.0 ^b^ (29.4–35.6)	35.4 ^b^ (27.4–43.4)	33.4 ^b^ (25.0–41.7)
< 6 h a day	7.1 ^a^ (5.9–8.3)	13.3 ^e^ (9.2–17.5)	13.8 ^e^ (9.7–17.9)	18.9 ^b^ (12.8–25.0)	12.9 (7.0–18.7)	3.7 (2.8–4.6)	14.4 ^b^ (9.7–19.2)	12.4 ^b^ (8.3–16.4)	13.5 ^b^ (7.7–19.2)	10.1 ^d^ (4.8–15.4)
Reports trying to get enough sleep to feel well-rested	75.1 ^a^ (73.1–77.1)	68.8 ^d^ (63.3–74-2)	63.1 ^b^ (57.5–68.6)	69.5 (62.3–76.7)	67.8 (59.7–75.9)	67.6 (65.4–69.8)	53.8 ^b^ (47.0–60.6)	56.4 ^b^ (50.5–62.4)	70.5 (63.1–77.9)	54.2 ^e^ (45.3–63.1)

Estimates for smoking status and weight categories are based on EHR data from a very large cohort of adults who were KPNC members during all of calendar year 2016. Estimates for other behaviors are based on pooled data from the 2014/2015 and 2017 Member Health Surveys weighted to the age-gender composition of KPNC adult members in the relevant race/ethnic group whose preferred spoken language was English. All estimates are standardized to the age distribution of 65–79 year old adults (using age categories 65–69, 70–74, 75–79) in the U.S. population in 2016

Estimates are based on data from the 2014/2015 and 2017 Member Health Surveys weighted to the age-gender composition of the relevant race/ethnic group in KPNC in 2016 and then standardized to the age distribution of the U.S. population in 2016

^1^ White, Black, and Latinx adults: Healthy weight = BMI 18.5- < 27.5; Overweight or obese = BMI ≥ 27.5; Overweight = BMI 27.5 - < 30.0; Obese = BMI ≥ 30.0; Filipino and Chinese adults: Healthy weight = BMI 18.5 - < 23.0; Overweight or obese = BMI ≥ 23.0; Overweight = BMI 23 - < 27.5; Obese = BMI ≥ 27.5

^2^ Recommended limits: ≤ 1 drink per day for all women and for men aged ≤ 65; ≤ 2 drinks per day for men > age 65

^3^ Based on weighted pooled data from the 2015 and 2017 surveys

^a^ Within racial/ethnic group, women significantly different from men at *p* < .05

^b^ Significantly different from Whites at *p* < .001

^c^ Filipinos significantly different from Chinese at *p* < .05

^d^ Significantly different from Whites at *p* < .05

^e^ Significantly different from Whites at *p* < .01

^f^ Lower bound of 95% CI is < 0

**Table 4:** Summary of differences in weight and health behavior risks, White, Black, Latinx, Filipino, and Chinese women and men aged 35–64 years and 65–79 years

Weight and Health Behavior Risks	Women 35–64 yr		Men 35–64 yr		Women 65–79 yr		Men 65–79 yr	
**Weight**								
Healthy weight	BLF ↓ W; C ↑ W	F ↓ C	BLF ↓ W	F ↓ C	BLF ↓ W; C ↑ W	F ↓ C	LF ↓ W; C ↑ W	F ↓ C
Overweight or obese	BLF ↑ W; C ↓ W	F ↑ C	BLF ↑ W	F ↑ C		F ↑ C		F ↑ C
Obese	BLF ↑ W; C ↓ W	F ↑ C	BL ↑ W; FC ↓ W	F ↑ C	BL ↑ W; FC ↓ W	F ↑ C	BLF ↑ W; C ↓ W	F ↑ C
**Smoking**								
Never smoker	LFC ↑ W	F ↓ C	LC ↑ W; F ↓ W	F ↓ C	LFC ↑ W	–	BF ↓ W; C ↑ W	F ↓ C
Current smoker	B ↑ W; LFC ↓ W	F ↑ C	BF ↑ W; LC ↓ W	F ↑ C	B ↑ W; LFC ↓ W	F ↑ C	B ↑ W; C ↓ W	F ↑ C
**Alcohol use**								
Stays within recommended limits	BFC ↑ W	–	BC ↑ W	F ↓ C	BLFC ↑ W	–	L ↓ W; FC ↑ W	F ↓ C
**Dietary behaviors**								
Fruit and vegetable consumption:								
≥ 3 servings a day	BLFC ↓ W	F ↓ C	BLFC ↓ W	F ↓ C	BLF ↓ W	F ↓ C	BLFC ↓ W	F ↓ C
≥ 5 servings a day	BLFC ↓ W	F ↓ C	BLF ↓ W	F ↓ C	BLF ↓ W	F ↓ C	BLFC ↓ W	F ↓ C
Usually tries to avoid high sodium/high salt foods	BLF ↓ W	F ↓ C	F ↓ W	–	LF ↓ W	–	–	–
Frequency consumes sugar-sweetened beverages:								
< Once a week or never	BLF ↓ W	F ↓ C	BLF ↓ W	F ↓ C	BLFC ↓ W	–	BLF ↓ W	–
≥ 3 days a week	BLF ↑ W	F ↑ C	BLF ↑ W	F ↑ C	BLF ↑ W	F ↑ C	BLF ↑ W	–
≥ 5 days a week	BLF ↑ W	F ↑ C	BLF ↑ W	F ↑ C	BLF ↑ W	–	BLF ↑ W	–
Reports trying to eat mostly healthy foods	BLF ↓ W	–	–	–	–	–	–	–
**Exercise/physical activity frequency**								
≥ 3 days a week	BFC ↓ W	–	C ↓ W	–	BL ↓ W; C ↑ W	F ↓ C	–	–
≥ 5 days a week	BLF ↓ W	F ↓ C	LC ↓ W	–	BLF ↓ W	F ↓ C	B ↓ W	–
< once a week	B ↑ W	–	BC ↑ W	–	FC ↓ W	–	–	–
Reports trying to get exercise most days	F ↑ W	F ↑ C	BF ↑ W	F ↑ C	FC ↑ W	–	LF ↑ W	–
**Usual amount of sleep**								
≥ 7 h a day	BLFC ↓ W	F ↓ C	BLF ↓ W	F ↓ C	BLFC ↓ W	–	BLFC ↓ W	–
< 7 h a day	BLFC ↑ W	F ↑ C	BLF ↑ W	F ↑ C	BLFC ↑ W	–	BLFC ↑ W	–
< 6 h a day	BLFC ↑ W	–	BLF ↑ W	F ↑ C	BLF ↑ W	–	BLFC ↑ W	–
Reports trying to get enough sleep to feel well-rested	BLC ↓ W	–	BC ↓ W	–	BL ↓ W	–	BLC ↓ W	F ↑ C

*W* White, *B* African American/Black, *L* Latinx, *F* Filipino, *C* Chinese; ↑: age-standardized prevalence significantly (*p* < .05) higher than comparison racial/ethnic group;.↓: age-standardized prevalence significantly (*p* < .05) lower than comparison racial/ethnic group; when racial/ethnic groups are not listed in a comparison with Whites, this indicates they did not significantly differ from the comparison racial/ethnic group; − : indicates that Filipinos did not significantly differ from Chinese

### Weight

#### Race/ethnicity

Based on standard BMI weight ranges, in both age groups, Black, Latina, and Filipina women were less likely, and Chinese women more likely than White women to be in the healthy weight range. Black, Latina, and Filipina women were more likely, and Chinese women less likely than White women to be in the overweight/obese and obese ranges. In the middle-aged group, Black, Latino, and Filipino men were less likely to be in the healthy weight range and more likely to be in the overweight/obese and obese ranges than White men. The percentages of Chinese adults within healthy weight and overweight/obese weight ranges were similar to those for White men, but Chinese men were significantly less likely to be in the obese range. In the older-aged group, Latino and Filipino men were less likely than White men to be in the healthy weight range and more likely to be in the overweight/obese range, while Black and Chinese men did not significantly differ from White men. Older-aged Black, Latino, and Filipino men were more likely, and Chinese men less likely than White men to be in the obese range. In both age groups, Filipino women and men were less likely than Chinese women and men to be in the healthy weight range and more likely to be in the overweight/obese and obese ranges.

#### Gender

In the middle-aged group, White, Latina, Filipina, and Chinese women were more likely than their male counterparts to be in the healthy weight range, and less likely to be in the overweight/obese and obese ranges. Middle-aged Black women were more likely than men to be in the obese range. In the older-aged group and in all racial/ethnic groups, women were more likely than men to be in the healthy weight range and less likely to be in the overweight/obese range. However, Black women were more likely and Filipina and Chinese women less likely than their male counterparts to be in the obese range, with no significant gender difference in the White and Latinx groups.

#### Age group

The percentages of women in the healthy weight and overweight/obese ranges did not significantly differ by age group across all racial/ethnic groups. However, Black, Latina, and Filipina women in the middle-aged group were more likely to have obesity than their female counterparts in the older-aged group. In all racial/ethnic groups, middle-aged men were less likely than men in the older-aged group to be in the healthy weight range and more likely to be in the overweight/obese and obese ranges.

### Smoking status

#### Race/ethnicity

Among women in both age groups, Black women were more likely and Latina, Filipina, and Chinese women less likely than White women to be current smokers. In both age groups, Latina, Filipina and Chinese women were more likely than White women to be never smokers, while Black women did not significantly differ from White women. Among men in the middle-aged group, Black and Filipino men were more likely and Latino and Chinese men were less likely than White men to be current smokers. Latino and Chinese men were more likely and Filipino men less likely to be never smokers. In the older-aged group, Black men were more likely and Chinese men less likely than White men to be current smokers, with no significant difference between Latino, Filipino, and White groups. Black and Filipino men were less likely and Chinese men more likely than White men to be never smokers, while Latinos did not significantly differ from White men. In both age groups, Filipino men and women were more likely than Chinese women and men to be current smokers and less likely to be never smokers.

#### Gender

In both age groups and across all racial/ethnic groups, women were less likely than men to be current smokers and more likely to be never smokers.

#### Age group

Middle-aged White, Black and Filipino women and men in all five racial/ethnic groups were more likely than older-aged adults in these same subgroups to be current smokers. Middle-aged men in all racial/ethnic groups and middle-aged women in all but the Filipina group were more likely than those in the older-aged group to be never smokers.

### Alcohol use

#### Race/ethnicity

Black, Filipina, and Chinese women in both age groups and Latina women in the older-aged group were more likely than similarly aged White women to be non-drinkers or to stay within the recommended ≤ 1 drink a day limit. Among middle-aged men, Black and Chinese men were more likely and Latino men less likely than White men to stay within the recommended ≤ 2 drink a day limit. In the older-aged group, Filipino and Chinese men were more likely than White men to stay within the recommended ≤ 1 drink limit, with percentages for Black and Latino men similar to Whites. In both age groups, percentages of Filipino and Chinese men and women who kept their alcohol use within recommended daily limits did not significantly differ.

#### Gender

In all racial/ethnic and age groups with the exception of middle-aged Filipinos, women were more likely than men to stay within their recommended daily alcohol intake limit.

#### Age group

Across all racial/ethnic groups, middle-aged women were less likely than older-aged women to stay within the ≤ 1 drink a day limit. Middle-aged White, Black, Latino, and Chinese men were more likely than men in the older-aged group to stay within the age-specific recommended daily alcohol intake limit, with no difference among Filipino men. However, the recommended limit for men > age 65 is 2 drinks a day, whereas it is only 1 drink a day for men ≤ age 65. The percentages of older-aged men who stayed within ≤2 drinks a day (83.4% of White, 82.0% of Black, 78.9% of Latino, 87.9% of Filipino, and 94.3% of Chinese men) were very similar to those in the middle-aged group.

### Daily servings of fruits and vegetables

#### Race/ethnicity

White women and men in both age groups were significantly more likely than women and men in the other racial/ethnic groups to report consuming ≥3 servings of fruits and vegetables a day with one exception. In the older-aged group, White women and Chinese women were similar in percentages who consumed ≥3 servings of fruits and vegetables a day. Fruit and vegetable intake at this level was lowest among Filipino men and women in both age groups. While approximately 25 and 20% of middle-aged and older-aged White women, respectively, reported consuming ≥5 servings of fruits and vegetables daily, less than 15% of adults in other subgroups consumed at this level. Filipinas in both age groups and middle-aged Filipino men were significantly less likely than their Chinese age-specific counterparts to consume ≥3 servings of fruits and vegetables a day.

#### Gender

With the exception of Filipinos, in both age groups, there was a ≥ 10 point difference between the percentages of women who consumed ≥3 servings of fruits and vegetables a day compared to men in the same racial/ethnic and age group. White and Latina women in both age groups and Black women in the older-aged group were more likely than men to consume ≥5 servings a day.

#### Age group

Middle-aged White women and Latino men were more likely to consume ≥3 and ≥ 5 servings of fruits and vegetables a day than those in the older-aged group, and middle-aged White men were more likely than older-aged White men to consume ≥3 servings a day.

### Avoidance of high sodium/high salt foods

#### Race/ethnicity

In the middle-aged group, Latina and Filipina women and Filipino men were less likely than their White counterparts to indicate that they tried to avoid high sodium/high salt foods most or all of the time. In the older-aged group, Latina and Filipina women were less likely than White women to engage in this behavior, with no racial/ethnic differences seen among men. Middle-aged Filipinas were less likely than middle-aged Chinese women to try to avoid high sodium foods most or all of the time, but there was no significant difference in the older-aged group. There was also no difference between Filipino and Chinese men in sodium avoidance in either age group.

#### Gender

In the middle-aged group, White, Latina, and Chinese women were more likely than men to try to avoid high sodium foods most or all of the time. In the older-aged group, there was only a gender difference among White adults.

#### Age group

Middle-aged Black women and men and middle-aged White men were less likely than those in the older-aged group to indicate that they try to avoid high sodium foods most or all of the time.

### Consumption of sugar-sweetened beverages (SSBs)

#### Race/ethnicity

In both age groups, Black, Latinx, and Filipino men and women were significantly more likely than Whites to consume SSBs ≥5 days a week and ≥ 3 days a week, and were significantly less likely than Whites to consume SSBs < once a week or never. Frequency of consumption among Chinese adults did not differ from White adults except among older-aged women, where lower percentages of Chinese women indicated consumption at the < once a week or never level. Filipina women in both age groups and Filipino men in the middle-aged group were more likely to consume SSBs ≥3 days a week than similarly aged Chinese adults, and in the middle-aged group, Filipina women and men were less likely than Chinese adults to consume SSBs < once a week or never.

#### Gender

White and Latina women in both age groups and Filipina women in the middle-aged group were less likely than men to consume SSBs ≥3 days a week. In the middle-aged group, across all racial/ethnic groups, women were more likely than men to consume SSBs < once a week or never, but this gender differences was only observed among White and Latinx adults in the older-aged group.

#### Age group

Middle-aged White and Latinx women and men and Filipino men were less likely than those in the older-aged group to consume SSBs < once a week or never. No significant age group differences were observed for SSB consumption at the ≥3 and ≥ 5 days a week levels.

### Effort to eat mostly healthy foods

#### Race/ethnicity

Middle-aged Black, Latina, and Filipina women were less likely than similarly aged White women to say they try to eat healthy foods, with no significant racial/ethnic differences observed among middle-aged men and among older-aged adults. Among most of the racial/ethnic groups, over 20% of women and approximately 30% of men did not endorse an effort to eat mostly healthy foods. Filipino adults did not significantly differ from Chinese adults on this measure.

#### Gender

In both age groups, White and Chinese women were more likely than their male counterparts to report trying to eat healthy foods.

#### Age group

No significant age group differences were observed.

### Exercise/physical activity

#### Race/ethnicity

Black, Filipina, and Chinese women in the middle-aged group and Black and Latina women in the older-aged group were less likely than similarly aged White women to get exercise or physical activity ≥3 days a week. In the middle-aged group, Black women and Black and Chinese men were more likely than Whites to report getting exercise less than once a week, and in the older-aged group, Filipina and Chinese women were less likely than White women to get exercise less than once a week, but no racial/ethnic differences were observed among men. In the middle-aged group, Filipino women and men were more likely than Chinese women and men to say they tried to get exercise most days, although this was not reflected in differences in reported exercise frequency.

#### Gender

In both groups, Black and Latina women, and in the older-aged group, White women, were less likely than men to get exercise ≥3 days and ≥ 5 days a week, but did not differ at < once a week. In the older-aged group, Filipina women were less likely than Filipino men to report getting exercise ≥5 days a week, and Latina women were less likely than Latino men to make an effort to exercise most days.

#### Age group

Middle-aged White, Latino, and Chinese men and middle-aged Filipina and Chinese women were less likely than those in the older-aged group to report getting exercise or physical activity ≥3 days a week, and middle-aged White and Chinese women and men were less likely than those in the older-aged group to report getting exercise ≥5 days a week. Middle-aged White women were significantly but only slightly less likely than older-aged White women to report getting exercise < once a week. Middle-aged White, Black and Latina women were more likely than the older-aged group to say they tried to get exercise most days, while middle-aged Chinese women and Latino men were less likely than those in the older-aged group to report making this effort.

### Sleep

#### Race/ethnicity

White women and men were more likely to report getting ≥7 h of sleep a day than Black, Latinx, and Filipino women and men in both age groups, Chinese women in both age groups, and Chinese men in the older-aged group. Black, Latinx, and Filipino women and men in both age groups, middle-aged Chinese women, and older-aged Chinese men were more likely than White adults to indicate getting < 6 h of sleep a day. Black adults, Latina women, and Chinese men in both age groups were less likely than similarly aged White adults to say that they made an effort to get enough sleep to feel well rested. In the middle-aged group, Filipino adults were less likely than Chinese adults to get ≥7 h of sleep a day, and middle-aged Filipino men were more likely than Chinese men to say they got < 6 h of sleep a day.

#### Gender

In the middle-aged group, White women were more likely than White men to get ≥7 h of sleep a day, while the opposite was true among White adults in the older-aged group. In both age groups, White women were more likely than White men to report trying to get enough sleep to feel well rested. No gender differences on the sleep behaviors were seen in the other racial/ethnic groups.

#### Age group

Compared to those in the older-aged group, middle-aged Black women and White, Black, Latino, and Filipino men were less likely to get ≥7 h of sleep a day. Black women and men and White men in the middle-aged group were more likely than those in the older-aged group to get < 6 h of sleep. Middle-aged Black and Latino men were more likely than those in the older-aged group to say that they tried to get enough sleep to feel well-rested, despite being less likely to report getting the recommended ≥7 h of sleep a day.

### Health behaviors among adults who report trying to engage in healthy behaviors

As shown in Table 5, in subgroups of adults who indicated that they were trying to eat healthy, get daily exercise most days, and get enough sleep to feel well-rested, we observed patterns of racial/ethnic differences in dietary, exercise, and sleep behaviors and gender differences in fruit and vegetable and sugar-sweetened beverage consumption similar to those found in the overall study population. For example, among those who reported trying to eat mostly healthy foods, Black, Latinx, Filipino, and Chinese women and men were significantly less likely than White women and men to consume ≥3 and ≥ 5 servings of fruit/vegetables a day. Similarly, among those who reported trying to get exercise most days, Black, Latinx, Filipino, and Chinese adults were less likely than White adults to get exercise ≥3 days a week, and all but Chinese women were less likely than White adults to get exercise ≥5 days a week. Across all racial/ethnic groups, much larger percentages of adults reported that they were trying to eat healthy, get exercise most days, and get enough sleep to feel well-rested than was reflected in the actual dietary, exercise, and sleep behaviors they reported.

**Table 5:** Differences in health behaviors among White, Black, Latinx, Filipino, and Chinese women and men aged 35–79 years who say they are trying to eat healthy, get exercise most days, and get enough sleep to feel well rested

Health behaviors	Women					Men				
	White	Black	Latina	Filipina	Chinese	White	Black	Latino	Filipino	Chinese
	% (95% CI)	% (95% CI)	% (95% CI)	% (95% CI)	% (95% CI)	% (95% CI)	% (95% CI)	% (95% CI)	% (95% CI)	% (95% CI)
**Reports trying to eat mostly healthy foods**										
Fruit and vegetable intake:										
≥ 3 servings a day	72.5 ^a^ (70.9–74.1)	46.8 ^ab^ (42.9–50.7)	45.9 ^ab^ (42.7–49.1)	30.0 ^bc^ (25.5–34.4)	58.0 ^ab^ (52.9–63.0)	53.8 (51.7–55.8)	25.7 ^b^ (21.5–29.8)	32.4 ^b^ (29.0–35.8)	23.0 ^bc^ (17.8–28.2)	42.3 ^b^ (36.7–48.0)
≥ 5 servings a day	27.5 (25.9–29.1)	13.3 ^ab^ (10.6–16.0)	14.5 ^ab^ (12.2–16.7)	7.3 ^b^ (4.7–10.0)	16.1 ^b^ (12.3–19.8)	17.8 (16.2–19.5)	5.9 ^b^ (3.7–8.1)	7.6 ^b^ (5.6–9.5)	3.8 ^bc^ (1.5–6.2)	14.2 (10.2–18.3)
Tries to avoid high sodium/high salt foods most or all of the time	73.0 ^a^ (71.4–74.6)	68.5 ^b^ (64.9–72.2)	68.7 ^ab^ (65.7–71.7)	59.0 ^bc^ (54.2–63.8)	66.2 ^b^ (61.4–71.1)	65.7 (63.7–67.7)	67.5 (63.2–71.9)	64.1 (60.6–67.6)	55.8 ^b^ (49.8–61.8)	61.3 (55.7–66.9)
Frequency consumes sugar-sweetened beverages:										
< Once a week or never	66.8 ^a^ (64.5–69.1)	39.7 ^ab^ (35.3–44.1)	55.3 ^ab^ (51.6–59.0)	51.4 ^abc^ (45.3–57.6)	59.5 ^ab^ (53.2–65.8)	53.9 (51.2–56.6)	32.0^b^ (26.9–37.1)	38.0 ^b^ (26.6–34.4)	35.6 ^bc^ (28.1–43.2)	46.5 ^b^ (39.5–53.5)
≥ 3 days a week	21.0 ^a^ (19.0–23.0)	43.8 ^b^ (39.3–48.3)	29.3 ^ab^ (25.8–32.7)	35.3 ^abc^ (29.5–41.2)	22.6 ^a^ (17.3–27.9)	28.6 (26.1–31.0)	46.3^b^ (40.9–51.8)	42.2 ^b^ (38.1–45.4)	49.3 ^bc^ (41.4–57.2)	32.7 (26.1–39.2)
≥ 5 days a week	16.2 ^a^ (14.4–18.0)	29.3 ^b^ (25.1–33.4)	22.7 ^ab^ (19.6–25.9)	29.2 ^bc^ (23.6–34.8)	16.7 (12.0–21.5)	22.3 (20.0–24.6)	32.3 ^b^ (27.2–37.5)	30.5 ^b^ (26.6–34.4)	36.7 ^bc^ (29.0–44.8)	22.5 (16.7–28.2)
**Reports trying to get exercise most days**										
Gets exercise ≥3 days a week	94.9 (94.0–95.8)	88.0 ^b^ (85.2–90.8)	89.3 ^b^ (87.2–91.4)	82.9 ^b^ (79.2–86.6)	89.5 ^b^ (86.0–92.9)	93.5 (92.3–94.6)	88.1 ^b^ (85.0–91.2)	90.9 ^b^ (88.7–93.0)	83.7 ^b^ (79.3–88.1)	87.5 ^b^ (83.5–91.4)
Gets exercise ≥5 days a week	59.9 (57.9–61.8)	42.3 ^ab^ (38.1–46.5)	49.5 (46.1–53.0)	40.9 ^bc^ (36.1–45.7)	57.0 (51.5–62.5))	62.4 (60.3–64.6)	50.8 ^b^ (46.1–55.5)	53.7 ^b^ (50.0–57.4)	49.0 ^b^ (43.2–54.8)	51.0 ^b^ (45.3–56.7)
**Reports trying to get enough sleep to feel well-rested**										
Typically gets ≥7 h of sleep a day	74.9 (73.3–76.5)	52.0 ^b^ (47.8–56.1)	63.7 ^b^ (60.3–67.0)	51.4 ^bc^ (46.4–56.4)	65.0 ^b^ (59.8–70.2)	76.0 (74.1–77.9)	49.3 ^b^ (44.3–54.4)	61.5 ^b^ (57.8–65.3)	54.0 ^bc^ (47.8–60.2)	71.8 (66.2–77.3)
< 7 h of sleep a day	25.1 (23.5–26.7)	48.0 ^b^ (43.9–52.2)	36.4 ^b^ (33.0–39.7)	48.6 ^bc^ (43.6–55.6)	35.0 ^b^ (29.8–40.2)	24.0 (22.2–25.9)	50.7 ^b^ (45.6–55.7)	38.5 ^b^ (34.7–42/2)	46.0 ^bc^ (39.8–52.2)	28.3 (22.7–33.8)
< 6 h of sleep a day	5.7 (4.8–6.5)	19.6 ^b^ (16.3–222.9)	12.3 ^b^ (10.1–14.6)	17.7 ^bc^ (13.8–21.5)	10.9 ^b^ (7.4–14.4)	4.9 (3.9–5.9)	18.1 ^b^ (14.2–22.0)	11.7 ^b^ (9.2–14.2)	13.6 ^bc^ (9.4–17.8)	7.9 (4.7–11.0)

Estimates are based on pooled data from the 2014/2015 and 2017 Member Health Surveys weighted to the age-gender composition of the relevant race/ethnic group in KPNC in 2016 and then standardized to the age distribution of adults aged 35–79 in the U.S. population in 2016. Consumption of sugar-sweetened beverages is based on weighted pooled data from the 2015 and 2017 Member Health Surveys. White: non-Hispanic White; Black: African American/Black

^a^ Significantly different from men in same racial/ethnic group at *p* < .05

^b^ Significantly different from Whites at *p* < .05

^c^ Filipinos significantly different from Chinese at *p* < .05

### Health behaviors among adults with diabetes and/or hypertension

Table 6 shows that the same pattern of racial/ethnic and gender differences found in the larger study population were also observed in the group of adults with diabetes and/or hypertension. Separate prevalence data are shown for women and men only for those health behaviors where significant gender differences were found in at least two racial/ethnic groups. Among both women and men, Black, Latinx, Filipino and Chinese adults were less likely than White adults to eat ≥3 servings of fruits and vegetables a day, and in all but the Filipino group, women were more likely than men to consume ≥3 servings. As a group, Filipino adults were less likely to consume ≥3 servings than Chinese adults, but the difference was only statistically significant among women. Black, Latinx, and Filipino women and men were less likely than White adults to consume SSBs < once a week or never and were more likely to consume SSBs ≥3 days a week. Chinese women were less likely than White women to consume SSBs < once a week or never, but SSB consumption by Chinese adults did not otherwise differ from that of White adults. Significant gender differences in SSB consumption were

 only observed for White and Latinx adults, with women being more likely than men to consume SSBs < once a week and less likely to consume SSBs ≥3 days a week. Latinx and Filipino adults were also less likely than White adults to try to avoid high sodium/high salt foods most or all of the time, with no significant differences by gender observed. Again, there were significant gender differences within racial/ethnic groups with regard to fruit and vegetable and SSB consumption.

**Table 6:** Differences in dietary- exercise- and sleep behaviors among White, Black, Latinx, Filipino, and Chinese adults aged 35–79 with diabetes and/or high blood pressure

Health behaviors	White	Black	Latinx	Filipino	Chinese
	% (95% CI)	% (95% CI)	% (95% CI)	% (95% CI)	% (95% CI)
**Dietary behaviors**					
Usually eats ≥3 servings of fruits and vegetables a day	50.8 ^a^ (49.0–52.6)	31.6 ^ab^ (28.3–34.9)	33.6 ^ab^ (30.3–37.0)	19.0 ^bc^ (15.5–22.6)	37.8 ^ab^ (31.8–43.7)
Women	61.0 ^a^ (58.4–63.5)	40.3 ^ab^ (35.8–44.8)	38.2 ^ab^ (33.3–43.1)	21.6 ^bc^ (16.6–26.6)	51.9 ^a^ (43.1–60.7)
Men	41.0 (38.5–43.5)	19.3 ^b^ (15.1–23.5)	29.6 ^b^ (25.0–.34.2)	16.0 ^b^ (11.0–21.0)	23.2 ^b^ (16.0–30.4)
Tries to avoid high sodium/high salt food most or all of the time	59.1 (57.3–60.9)	59.6 (56.2–63.0)	53.0 ^b^ (49.5–56.5)	48.8 ^b^ (44.3–53.3)	55.2 (49.1–61.4)
Frequency consumes sugar-sweetened beverages					
< Once a week or never	59.0 ^a^ (56.6–61.4)	33.9 ^b^ (30.1–37.7)	43.9 ^ab^ (39.8–48.0)	42.8 ^b^ (37.1–48.5)	47.0 ^b^ (39.4–54.6)
Women	64.3 ^a^ (60.9–67.6)	35.5 ^b^ (30.4–40.5)	54.3 ^ab^ (48.5–60.1)	46.9 ^b^ (39.2–54.5)	48.5 ^b^ (37.3–59.7)
Men	53.9 (50.4–57.3)	31.8 ^b^ (26.1–37.5)	34.4 ^b^ (29.0–39.9)	37.8 ^b^ (29.4–46.3)	45.4 (35.1–55.6)
≥ 3 days a week	28.0 ^a^ (25.8–30.3)	48.2 ^b^ (44.2–52.3)	41.0 ^ab^ (36.9–45.0)	44.3 ^b^ (38.6–50.0)	36.6 (29.2–43.9)
Women	24.4 (21.3–27.4)	47.0 ^b^ (41.7–52.3)	32.3 ^b^ (26.8–37.7)	39.6 ^b^ (32.7–47.1)	33.5 (23.0–44.0)
Men	31.6 (28.4–34.8)	49.9 ^b^ (43.7–56.1)	49.0 ^b^ (43.2–54.7)	50.0 ^b^ (41.3–58.8)	39.8 (29.6–50.0)
**Exercise frequency**					
≥ 3 days a week	71.8 (70.1–73.4)	66.6 ^b^ (63.3–69.9)	68.6 (65.3–71.9)	70.3 (66.1–74.5)	71.9 (66.3–77.6)
< once a week	13.7 (12.5–15.0)	15.7 (13.2–18.3)	14.9 (12.4–17.4)	12.2 (9.1–15.2)	9.7 (6.0–13.3)
**Usual amount of sleep**					
≥ 7 h a day	66.5 (63.8–69.1)	47.9 ^b^ (44.1–51.8)	59.1 ^b^ (55.1–63.0)	47.3 ^b^ (42.2–52.4)	53.5 ^b^ (45.4–61.5)
< 7 h a day	33.6 (30.9–36.2)	52.1 ^b^ (48.2–56.0)	40.9 ^b^ (37.0–44.9)	52.7 ^b^ (47.7–57.8)	46.6 ^b^ (38.5–54.6)
< 6 h a day	8.3 (7.3–9.4)	22.8 ^b^ (19.8–25.8)	15.0 ^b^ (12.4–17.5)	20.1 ^b^ (16.4–23.8)	13.1 ^b^ (8.8–17.3)

Estimates are based on pooled data from the 2014/2015 and 2017 Member Health Surveys weighted to the age-gender composition of the relevant race/ethnic group in KPNC in 2016 and then standardized to the age distribution of adults aged 35–79 in the U.S. population in 2016. Sugary drink consumption is based on weighted pooled data from the 2015 and 2017 Member Health Surveys. Separate estimates are shown for women and men only when there are statistically significant gender differences

^a^ Significantly different from men in same racial/ethnic group at *p* < .05

^b^ Significantly different from Whites at *p* < .05

^c^ Filipinos significantly different from Chinese at *p* < .05

Across all of the racial/ethnic groups, approximately 2/3 of adults in this group reported getting exercise ≥3 days a week, with Black adults being the only group with a significantly lower prevalence than Whites and no significant gender differences within racial/ethnic groups. With regard to sleep, Black, Latinx, Filipino and Chinese adults were less likely than White adults to get the recommended ≥7 h of sleep and more likely to get < 6 h of sleep a day. In this subgroup, Filipino adults did not differ from Chinese adults on any of the sleep behaviors.

## Discussion

In an earlier study of racial/ethnic differences in cardiometabolic risks in 45–84 year old members of this Northern California health plan, Black, Latinx, and Filipino women and men had higher prevalence of diabetes and hypertension than White and Chinese adults [7]. In our current study of adults in the same health plan population, we separated adults in these same racial/ethnic groups into middle-aged and older-aged groups and compared them on weight and cardiometabolic health behaviors. We found that in both age groups, Black, Latinx, and Filipino adults were more likely to have BMIs in the overweight and obese ranges than White and Chinese adults and were less likely to engage in health behaviors that prevent or help control cardiometabolic conditions. Specifically, both women and men in these three racial/ethnic groups reported lower fruit and vegetable intake, more frequent sugar-sweetened beverage consumption, and were less likely to meet recommended hours of sleep. Further, based on our EHR-derived smoking data, we found that Black adults and Filipino men (but not women) were more likely than White adults to be current smokers. Interestingly, we detected less variation across racial/ethnic groups in terms of exercise and physical activity frequency, especially among men.

Our results also underscore the importance of examining and reporting cardiometabolic health risks by gender and age group. Within racial/ethnic groups, we detected gender differences in current smoking, being in the healthy weight range, fruit and vegetable intake, and frequency of sugar-sweetened beverage consumption. However, we did not detect similar gender differences with regard to exercise/physical activity frequency and getting recommended hours of sleep. Racial/ethnic differences were larger among women than men, which may be attributed to men generally reporting poorer health behaviors than women within each racial/ethnic group, especially with regard to dietary practices like fruit and vegetable and sugary beverage consumption. Age group differences within gender and racial/ethnic group were primarily seen for exercise frequency and hours of sleep and not dietary behaviors, but these associations were not consistent across all racial/ethnic and gender subgroups.

Our findings also underscore the importance of disaggregating Asian health data by ethnic subgroup. Disaggregating data reveals ethnic-group differences in health risks, which are critical for the development of targeted recommendations regarding screening for and counseling about behavioral and biometric (weight, blood sugar, blood pressure) risk factors for cardiometabolic conditions. For example, our study found that Filipino adults reported engaging in overall more negative health behaviors than Chinese adults, and on many of our behavioral risk measures, Filipinos were more similar to African American/Black and Latinx adults than Chinese adults. The lower percentages consuming ≥3 servings of fruit and vegetables a day, higher frequency of consumption of sugar-sweetened beverages, and lower frequency of meeting recommended hours of sleep may underlie the higher prevalence of obesity found among Filipino adults compared to those found among Chinese adults. In turn, the higher prevalence of obesity combined with the higher prevalence of negative health behaviors may help explain the higher prevalence of diabetes and hypertension detected among Filipino compared to Chinese adults.

Several factors may underlie the racial/ethnic variation in reported health behaviors found in the current study. In particular, social determinants of health (SDOHs) like educational attainment, income, financial strain, and health literacy may be driving such racial/ethnic differences. Importantly, a previous study of SDOHs among middle-aged and older-aged Black, Latinx, Filipino, Chinese, and White adults in this same health plan based on contemporary Member Health Survey data identified racial/ethnic differences in SDOHs that may help explain why recommended health behaviors are more difficult to achieve for some populations [37]. For example, Black and Latinx adults were less likely to be college graduates than White, Filipino, and Chinese adults, and Black and Latinx adults in both age groups and Filipino adults in the older-aged group were more likely to be part of a lower income household than White and Chinese adults. Black and Latinx adults, but not Filipino adults, were also more likely than White adults to indicate that they ate less fruits and vegetables than they would have wanted to due to the cost. Additionally, middle-aged Black, Latinx, and Filipino adults and older-aged Latinx and Filipino adults were less likely than similarly-aged White adults to believe that their weight, diet, and exercise could have a large impact on their health, with Filipino adults being the least likely of all five racial/ethnic groups to believe that maintaining a healthy weight and healthy behaviors is important for good health. Although not formally tested, these racial/ethnic differences in SDOHs suggest that financial strain may be more of a factor influencing healthy eating among Black and Latinx adults, while lack of a strong belief in the importance of maintaining a healthy diet and healthy weight may be a more influential factor for Filipino adults.

Findings from this study yield three major implications for intervention efforts. First, given the high percentages of men in all of the racial/ethnic groups who reported unhealthy dietary behaviors, interventions that promote healthy eating among men are needed. Research suggests that compared to women, men tend to be both less knowledgeable about nutrition and less concerned about the adverse consequences of unhealthy eating until they have already developed a diet-related health condition [38, 39]. Previous interventions that have attempted to engage men have had limited success, with one systematic review finding that only one in four participants in behavioral weight loss programs were men [40]. More research is needed to identify intervention approaches that may be more appealing to men, for example, self-guided interventions rather than group- or individual-based counseling interventions [40]. Alternatively, there is some evidence that a focus on improving eating and food preparation behaviors of women partners can have a positive effect on men’s diet, since in many hetero-partnered households, the woman spouse/partner has primary responsibility for planning, purchasing and preparing food for the family. For example, a study of husbands of intervention group women in the Women’s Health Initiative had a modest but significant reduction in dietary fat compared to husbands of control group women, which researchers were able to attribute to the passive effect of husbands accepting or not noticing changes their wives had made in food purchases and preparation [41]. A qualitative study of middle-aged and older-aged African American married men found that while the husbands were often unhappy with the changes toward healthier foods and food preparation that their wives made, they were willing to accept the dietary changes to maintain harmony in their relationships and social roles [42].

Second, we found that weekly frequency of sugar-sweetened beverage consumption was relatively high across all groups, and especially high among Black, Latinx, and Filipino adults. This was observed not only among the overall study groups, but also among adults who reported that they were trying to eat healthy food and adults who had already been diagnosed with diabetes or hypertension. A growing body of research has associated consumption of sugar-sweetened beverages, especially those made with high fructose corn syrup with higher risk of obesity [43], type 2 diabetes [43], coronary heart disease [43], and hypertension [44–46]. There is also some evidence that sugar-sweetened beverages can increase risk of hypertension and diabetes through their contribution to a high dietary glycemic load, independent of the pathway through obesity [43]. Screening for and education about the harms of sugar-sweetened beverage consumption should thus be an important component of clinical care and public health campaigns to improve cardiometabolic and cardiovascular health, especially since sodas and other sugar-sweetened beverages can be very cheaply replaced by tap water.

Third, sleep is increasingly recognized as a risk factor for poor cardiometabolic health [47], and a number of evidence-based sleep interventions exist. Yet, relative to diet or physical activity, sleep risk (e.g., number of hours and quality of sleep) is less likely to be screened for routinely in primary care, and fewer interventions target sleep as a modifiable risk factor to improve cardiometabolic health [48]. Given the relatively low prevalence of adults in all racial/ethnic groups who met the recommended hours of sleep in our study, improving sleep may represent an under-tapped mechanism to improve cardiometabolic outcomes. Sleep interventions may be especially important for improving the health of populations who are at higher risk of experiencing sleep disturbance due to working in jobs that have irregular working hours. For example, in California, Filipinos alone make up 20% of the nursing workforce [49], and sleep complaints are common among front-line healthcare providers, especially those who work night shifts [50].

Our study has three major strengths. First, using data from two large data sources, we were able to examine eight different health behaviors relevant to cardiometabolic health. Second, we had sufficient racial/ethnic subgroup sample sizes to make explicit comparisons across gender and age groups within each racial/ethnic group. While the smaller numbers of Filipino and Chinese adults in the four age and gender subgroups led to lower power to detect differences between these groups, we were still able to show important differences in health behaviors between Filipino and Chinese adults that would have been masked if we had used data for an aggregated Asian racial group. Third, because the study data came from adults residing in a well-defined geographic area, we believe that our racial/ethnic comparisons are less biased by regional variation in health-related behaviors and lifestyle factors.

We also acknowledge that our study has limitations. Our survey response rate for middle-aged adults, and especially among Black, Latinx, and Filipino respondents in that age group, was relatively low. However, to adjust for potential non-response bias, our analyses were performed using respondent data weighted to reflect the age-gender composition of each racial/ethnic group within the health plan. Research suggests that weighting to demographic characteristics of the study population results in accurate estimates for most measures [51]. A second limitation is that health behaviors were based on self-report, thus there may be instances of under- or over-reporting of health behaviors. Third, although our racial/ethnic comparisons may be less biased by regional variation in health-related behaviors and lifestyle factors, results from this study may not generalize to other parts of California or other regions in the U.S. Fourth, because the survey was only conducted in English, our results cannot be generalized to non-English speaking adults who comprise sizable percentages of Latinx and Chinese adult populations in the U.S.. Finally, although our study provided a granular examination of racial/ethnic differences in weight and health behaviors (and gender and age group differences within each racial/ethnic group), the survey dataset did not enable us to provide a more detailed description of differences in types of foods and beverages consumed and types and quantity of exercise.

## Conclusion

In this study, we characterized and compared racial/ethnic, gender, and age groups on multiple behaviors relevant to cardiometabolic health using the same data source for adults in a single community-based insured population. We found that Black, Latinx, and Filipino adults, racial/ethnic groups with a documented higher prevalence of cardiometabolic conditions than adults of White and Chinese race/ethnicity, were more likely than White and Chinese adults in similar age-gender groups to have dietary and sleep behaviors associated with development and control of cardiometabolic conditions. Racial/ethnic and gender differences in these behaviors were found not only in the overall study population, but also in the subset of the population with diabetes and/or hypertension and in the subset of adults who reported they were trying to engage in health promoting behaviors. Further research is needed to elucidate details about dietary, exercise, and sleep behaviors and the social factors influencing these health behaviors and cardiometabolic health conditions among women and men in each of these racial/ethnic groups in order to develop culturally competent and effective clinical and public health approaches to reducing obesity and other cardiometabolic risks.

## Supplementary Information

**Additional file 1: Supplemental Table 1**. Unweighted base denominators and weighted age distributions for racial/ethnic subgroups- prior to age standardization^1^.

## Data Availability

The Kaiser Permanente Northern California (KPNC) Institutional Review Board has not provided approval for Member Health Survey data or variables derived from electronic health data to be placed in a public access repository. However, researchers can request access to use this study data by contacting the corresponding author (NPG) or the DOR Data Sharing Workgroup at DOR-DataSharingWorkgroup@kp.org.

## Abbreviations

AASM: American Association of Sleep Medicine
BMI: Body Mass Index
CMB: Cardiometabolic
DECKA2016: Demographically Enriched Cohort of Kaiser Adults 2016
EHR: Electronic health record
KPNC: Kaiser Permanente in Northern California
MHS: Member Health Survey
PA: Pennsylvania
SDOH: Social determinants of health
SRS: Sleep Research Society
SSB: Sugar-sweetened beverages
U.S.: United States
WHO: World Health Organization
