# Supplementary material for: Racial/ethnic, gender, and age group differences in cardiometabolic risks among adults in a Northern California health plan: a cross-sectional study
Source: BMC Public Health. 2021 Jun 25;21:1227. doi: 10.1186/s12889-021-11011-4 (PMC8235804; doi:10.1186/s12889-021-11011-4)
Supplement: Supplementary file 1 — Additional file 1: Supplemental Table 1. Unweighted base denominators and weighted age distributions for racial/ethnic subgroups- prior to age standardization1. [file 12889_2021_11011_MOESM1_ESM.docx]

| **Supplemental Table 1. Unweighted base denominators and weighted age distributions for racial/ethnic subgroups- prior to age standardization^1^** | | | | | | | | | | | |
| --- | --- | --- | --- | --- | --- | --- | --- | --- | --- | --- | --- |
|  | **Women** | | | | | **Men** | | | | | |
|  | **White** | **Black** | **Latina** | **Filipina** | **Chinese** | **White** | | **Black** | **Latino** | **Filipino** | **Chinese** |
| **For weight categories^2^** |  |  |  |  |  |  | |  |  |  |  |
| 35-64 years | N=229420 | N=39127 | N=63151 | N=36812 | N=23085 | N=206190 | | N=29661 | N=54687 | N=25658 | N=18288 |
| 35-44 years | 26.4% | 29.4% | 39.6% | 32.9% | 36.6% | 25.4% | | 26.1% | 37.4% | 30.7% | 34.0% |
| 45-54 years | 32.7% | 34.7% | 32.8% | 34.4% | 34.0% | 33.0% | | 36.2% | 34.8% | 34.5% | 33.3% |
| 55-64 years | 40.9% | 35.9% | 27.6% | 32.7% | 29.4% | 41.6% | | 37.7% | 27.8% | 33.3% | 32.7% |
| 65-79 years | N=111299 | N=12400 | N=13863 | N=10563 | N=6231 | N=96718 | | N=9304 | N=11632 | N=7746 | N=5973 |
| 65-69 years | 43.3% | 45.4% | 46.0% | 46.7% | 56.9% | 43.8% | | 47.6% | 44.9% | 47.1% | 54.6% |
| 70-74 years | 34.3% | 32.0% | 31.3% | 32.4% | 25.9% | 34.5% | | 30.4% | 32.9% | 32.3% | 25.3% |
| 75-79 years | 22.4% | 22.6% | 22.7% | 20.9% | 17.2% | 21.7% | | 22.0% | 22.2% | 20.6% | 20.1% |
| **For smoking status^2^** |  |  |  |  |  |  | |  |  |  |  |
| 35-64 years | N=324444 | N=57220 | N=86852 | N=49658 | N=32086 | N=289410 | | N=42272 | N=75900 | N=34755 | N=25415 |
| 35-44 years | 26.9% | 30.3% | 41.0% | 34.2% | 38.2% | 26.7% | | 27.7% | 39.1% | 32.4% | 35.1% |
| 45-54 years | 32.8% | 34.7% | 32.4% | 34.2% | 33.6% | 33.4% | | 36.3% | 34.6% | 34.6% | 34.0% |
| 55-64 years | 40.3% | 35.0% | 26.6% | 31.6% | 28.2% | 39.9% | | 36.0% | 26.3% | 33.0% | 30.9% |
| 65-79 years | N=147138 | N=17166 | N=17695 | N=13201 | N=7834 | N=123015 | | N=12248 | N=14320 | N=9625 | N=7315 |
| 65-69 years | 43.7% | 45.8% | 46.3% | 46.5% | 56.9% | 43.8% | | 47.3% | 45.4% | 47.1% | 54.6% |
| 70-74 years | 34.0% | 31.8% | 31.0% | 32.3% | 25.8% | 34.4% | | 30.6% | 32.3% | 31.9% | 25.3% |
| 75-79 years | 22.3% | 22.4% | 22.7% | 21.2% | 17.3% | 21.8% | | 22.1% | 22.4% | 21.1% | 20.1% |
| **For survey-based variables^3^** |  |  |  |  |  |  | |  |  |  |  |
| Ages 35-64 |  |  |  |  |  |  | |  |  |  |  |
| Sugary beverage consumption | N=1473 | N=468 | N=700 | N=248 | N=205 | N=1298 | | N=373 | N=586 | N=165 | N=208 |
| All other health behaviors | N=2617 | N=641 | N=971 | N=398 | N=340 | N=2237 | | N=492 | N=797 | N=267 | N=313 |
| 35-44 years | 26.9% | 30.4% | 41.2% | 34.4% | 38.5% | 27.1% | | 28.3% | 40.0% | 33.1% | 36.7% |
| 45-54 years | 32.8% | 34.6% | 32.2% | 34.2% | 33.6% | 33.4% | | 36.2% | 34.2% | 34.7% | 34.1% |
| 55-64 years | 40.3% | 35.0% | 26.6% | 31.4% | 27.9% | 39.5% | | 35.5% | 25.8% | 32.2% | 29.2% |
| Ages 65-79 |  |  |  |  |  |  |  | |  |  |  |
| Sugary drink consumption | N=1107 | N=205 | N=247 | N=113 | N=92 | N=1107 | N=169 | | N=252 | N=95 | N=101 |
| All other health behaviors | N=2086 | N=314 | N=359 | N=198 | N=140 | N=2103 | N=258 | | N=352 | N=173 | N=153 |
| 65-69 years | 43.8% | 46.05 | 46.5% | 46.6% | 57.1% | 44.1% | 47.5% | | 45.7% | 47.5% | 55.1% |
| 70-74 years | 34.0% | 31.7% | 31.0% | 32.3% | 25.8% | 34.3% | 30.5% | | 32.1% | 31.7% | 25.1% |
| 75-79 years | 22.2% | 22.4% | 22.5% | 21.1% | 17.1% | 21.6% | 22.0% | | 22.2% | 20.8% | 19.8% |
| ^1^ Age-standardization to the 2016 Census (American Community Survey) used the following distributions: For the 35-64 age group: age 35-44, 45.46%; age 45-54, 31.92%; age 55-64, 22.62%. For the 65-79 age group: age 65-69, 32.48%; age 70-74, 34.30%; age 75-79, 33.22%.  ^2^ Age distributions for the electronic health record-derived smoking status and weight category variables are based on actual (non-weighted) counts.   \| ^3^ Ns and age distributions for variables based on weighted survey data are approximations due to missing data. All sociodemographic characteristics and health behaviors, with the exception of sugary beverage consumption, are based on pooled respondent data from the 2014/2015 and 2017 cycles of the KPNC Member Health Survey that were weighted to the age-gender-race/ethnic distribution of the English speaking KPNC membership in 2016. A similarly constructed weighting factor was used for sugary beverage consumption, which only appeared in the was only included in the 2015 and 2017 surveys but is used with a weighting factor that adjusts the age distribution to that used for the other survey-based variables. \| \| --- \| | | | | | | | | | | | |
